# Multicenter retrospective study on the use of Curebest™ 95GC Breast for estrogen receptor-positive and node-negative early breast cancer

**DOI:** 10.1186/s12885-021-08778-5

**Published:** 2021-10-05

**Authors:** Fumine Tsukamoto, Koji Arihiro, Mina Takahashi, Ken-ichi Ito, Shozo Ohsumi, Seiki Takashima, Takaaki Oba, Masayuki Yoshida, Kazuki Kishi, Keisuke Yamagishi, Takayuki Kinoshita

**Affiliations:** 1 Department of Breast and Endocrine Surgery, Japan Community Health care Organization Osaka Hospital, Osaka, Japan; 2 Department of Anatomical Pathology, Hiroshima University Hospital, Hiroshima, Japan; 3 Department of Breast Oncology, National Hospital Organization Shikoku Cancer Center, Matsuyama, Japan; 4 Department of Surgery, Division of Breast and Endocrine Surgery, Shinshu University School of Medicine, Matsumoto, Japan; 5 Department of Pathology and Clinical Laboratories, National Cancer Center Hospital, Tokyo, Japan; 6 Sysmex Corporation, Kobe, Japan; 7 Division of Breast Surgery, National Hospital Organization Tokyo Medical Center, Tokyo, Japan

**Keywords:** ER-positive breast cancer, Curebest™ 95GC breast, DNA microarrays, Breast cancer staging, Node-negative breast cancer, Prognostic prediction

## Abstract

**Background:**

The benefits of postoperative chemotherapy in patients with estrogen receptor (ER)-positive breast cancer remain unclear. The use of tumor grade, Ki-67, or ER expression failed to provide an accurate prognosis of the risk of relapse after surgery in patients. This study aimed to evaluate whether a multigene assay Curebest™ 95GC Breast (95GC) can identify the risk of recurrence and provide more insights into the requirements for chemotherapy in patients.

**Methods:**

This single-arm retrospective multicenter joint study included patients with ER-positive, node-negative breast cancer who were treated at five facilities in Japan and had received endocrine therapy alone as adjuvant therapy. The primary lesion specimens obtained during surgery were analyzed using the 95GC breast cancer multigene assay. Based on the 95GC results, patients were classified into low-risk (95GC-L) and high-risk (95GC-H) groups.

**Results:**

The 10-year relapse-free survival rates were 88.4 and 59.6% for the 95GC-L and 95GC-H groups, respectively. Histologic grade, Ki-67, and PAM50 exhibited a significant relationship with the 95GC results. The segregation into 95GC-L and 95GC-H groups within established clinical factors can identify subgroups of patients using histologic grade or PAM50 classification with good prognosis without receiving chemotherapy.

**Conclusions:**

Based on the results of our retrospective study, 95GC could be used to evaluate the long-term prognosis of ER-positive, node-negative breast cancer. Even though further prospective validation is necessary, the inclusion of 95GC in clinical practice could help to select optimal treatments for breast cancer patients and identify those who do not benefit from the addition of chemotherapy, thus avoiding unnecessary treatment.

**Supplementary Information:**

The online version contains supplementary material available at 10.1186/s12885-021-08778-5.

## Background

Breast cancer is the most common cancer among women worldwide [1]. The overall survival and prevention of relapse after surgery are improving owing to the increase in the number of available treatment options. Estrogen receptor (ER)-positive breast cancers account for > 70% of all breast cancers and have favorable outcomes compared with ER-negative breast cancers [2]. Moreover, 10–20% of patients experience relapse after surgery [2, 3], and although chemotherapy improves breast cancer survival, its benefits in ER-positive breast cancers are being questioned lately [4].

In the past, the risk of breast cancer relapse was assessed by taking into account the patient’s age, tumor diameter, tumor grade, Ki-67, and ER expression. However, the risk of relapse and the need for chemotherapy are difficult to predict accurately based solely on clinicopathological factors.

Recently, it has been possible to more accurately predict recurrence risk in breast cancer patients by supplementing information from clinicopathological factors used in previous breast cancer multigene assays, with some already incorporated in clinical practice. Oncotype DX® (Exact Sciences Corporation, Madison, WI) is currently a widely used multigene classifier that can be used to stratify patients with node-negative ER-positive breast cancer into low-, intermediate-, and high-risk groups based on the expression of 21 genes [5–8]. Another widely used multigene assay is the MammaPrint® (Agendia, Inc., Amsterdam, Netherlands), which can analyze 70 genes to predict recurrence in patients with both ER-positive and ER-negative node-negative breast cancer. The PAM50 (NanoString Technologies, Inc., Seattle, WA) was developed to classify patients based on intrinsic subtypes, but it has also been used for recurrence prediction [9, 10]. The analysis of patient samples by various multigene assays revealed that risk categorization differs between tests [11].

Curebest™ 95GC Breast (95GC, Sysmex Corporation, Kobe, Japan) is a breast cancer multigene assay that uses DNA microarray to analyze the expression of 95 genes [12, 13] and has been available in Japan for research use only since 2013. In this assay, RNA is extracted from frozen or formalin-fixed paraffin-embedded tissue samples and the expression of 95 genes is analyzed by microarray. After this, the 95GC score is calculated from the expression of the 95 genes using a unique algorithm. By setting a specific cut-off value for the 95GC score, patients with ER-positive, node-negative breast cancer can be classified into low-risk (95GC-L) and high-risk (95GC-H) groups without the intermediate-risk group. According to a report by Naoi et al., in patients who received endocrine therapy alone as an adjuvant therapy, the recurrence rate was remarkably low in the low-risk group and significantly more favorable than that in the high-risk group, with 10-year recurrence-free survival rates of 93% for 95GC-L and 53% for 95GC-H [12]. From these results, owing to the extremely low risk of recurrence after surgery, adjuvant chemotherapy may be considered omissible in patients who are categorized as 95GC-L based on 95GC.

Thus far, the benefits of 95GC as a prognostic factor in patients with ER-positive, node-negative breast cancer have been evaluated only in retrospective studies conducted in a single facility or from public databases. Here, we aimed to assess the use of 95GC in patients from five Japanese facilities with ER-positive, node-negative breast cancer who received endocrine therapy alone as adjuvant therapy. To examine the relationships between 95GC and pathological factors associated with low reproducibility or inter-pathologist agreement, a repeat test was conducted with a central pathology review for Ki-67 and histologic grade and repeated analysis using array data for HER2.

This study aimed to evaluate relapse-free survival between the 95GC-L and 95GC-H groups and the differences in the clinical features of each group. It also aimed to analyze the outcome of 95GC regarding individual clinical features, such as histologic grade and intrinsic subtypes.

## Methods

### Study design

This non-interventional, non-invasive, and single-arm retrospective multicenter joint study included patients from five facilities in Japan (National Cancer Center Hospital, Shikoku Cancer Center, Shinshu University Hospital, Hiroshima University Hospital, and JCHO Osaka Hospital). The surgery was performed between January 19, 1999, and October 22, 2010, with a median follow-up period of 90 months. We followed the STROBE guidelines (checklist is included as Additional file 1).

Female patients aged ≥20 years with confirmed ER-positive invasive breast cancer with T1 or T2 primary lesions, no lymph node involvement, and no distant metastasis were included in the study. Patients who received adjuvant endocrine therapy regardless of drug classification, such as selective ER modulators (tamoxifen and toremifene), aromatase inhibitors (letrozole and exemestane), luteinizing hormone-releasing hormone agonist (goserelin and leuprolide), and a combination thereof (combination or sequential therapy) were also included. The primary lesion specimens were collected 30 min after surgery and stored at − 80 °C until analysis. Patients who received neoadjuvant therapy (prior to surgery) and adjuvant therapy other than endocrine therapy were excluded. In principle, only endocrine therapy is administered for T1/T2, lymph node-negative, and ER-positive breast cancer. Chemotherapy is recommended for patients with histologic grade 3, but it may not be administered at the patient’s request.

### Specimen standards

Frozen specimens from patients were delivered on dry ice from the participating facilities to Sysmex Corporation and cryosectioned using Tissue-Tek® O.C.T. compound (Sakura Finetek, Osaka, Japan). Sections measuring 6-μm thick were prepared from the top and bottom edges of the specimen and stained using hematoxylin and eosin, and the tumor cell percentage was calculated. The remaining specimen was reserved for later use for microarray measurement.

RNA was extracted and purified from the preserved specimens using the RNeasy® Lipid Tissue Mini Kit (Qiagen, Hilden, Germany). Only specimens that yielded > 17 ng/μL RNA, determined using the NanoDrop™ 2000 (Thermo Fisher Scientific Inc., Waltham, MA), and with RIN value > 5.0, calculated using the Agilent 2100 Bioanalyzer (Agilent Technologies, Santa Clara, CA), were considered valid for the study. Microarray measurements were performed using the GeneChip™ 3’IVT PLUS Reagent Kit (Thermo Fisher Scientific) and GeneChip™ Human Genome U133 Plus 2.0 Array (Thermo Fisher Scientific). All procedures were performed in accordance with the manufacturer’s protocol.

The microarray data obtained were evaluated for quality using MAS5 normalization, and only the data that complied with the following conditions were considered: GAPDH3’/5′ ratio < 3.0, average background value in the range of 20–100, scaling factor ≤ 15, percent *P* ≥ 30, spike-in labeling control lys_3_signal < phe_3_signal < dap_3_signal, and spike-in hybridization control BioB_3_signal < BioC_3_signal < BioD_3_signal < cre_3 signal. After normalizing the microarray data to analyze recurrence in patients using the RefRMA model, the 95GC algorithm was used to obtain the classification result of 95GC-L or 95GC-H, as described by Naoi et al. [12].

### Statistical analysis

Using the Kaplan–Meier method, the relapse-free survival curves of the 95GC-L and 95GC-H groups were plotted and evaluated using the log-rank test.

The correlation between clinical factors and 95GC was evaluated using Fisher’s exact test. The hazard ratio for recurrence was evaluated for each factor based on the Cox proportional hazard model, and multivariate analysis was performed using factors with statistically significant hazard ratios in the univariate analysis. Each clinical factor was analyzed, excluding the missing data values. Multivariate analysis only included patients with available data on all the evaluated factors. The sample size was determined by calculating the number of cases required to verify the predicted 5-year recurrence-free survival rate of Curebest 95GC Breast [12].

For patients with histologic grade 2 and those with the PAM50 luminal B subtype, relapse-free survival curves were evaluated for each 95GC risk group using the Kaplan–Meier method and log-rank test.

All statistical tests were performed using two-tailed tests, with a significance level of 5%. We used the following applications: for Fisher’s exact test, R ver. 4.0.2 (R Foundation for Statistical Computing, Vienna, Austria); for the Cox proportional hazard model, Kaplan-Meier method, and log-rank test, MedCalc ver. 12.7.0.0 (MedCalc Software Ltd., Ostend, Belgium).

## Results

### Patient characteristics

**Fig. 1:**
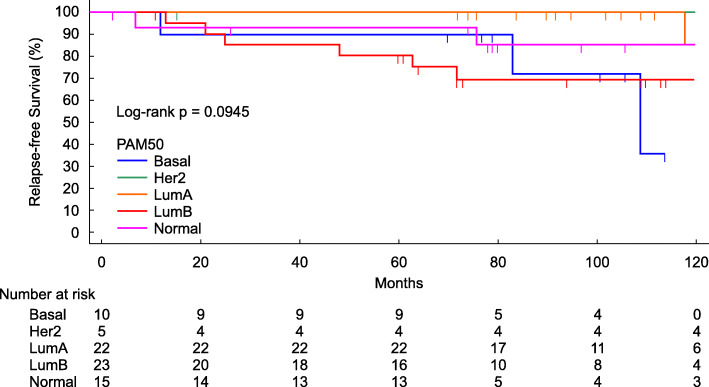
Relapse-free survival curves by PAM50 classification. Relapse-free survival curves were evaluated in patients according to the PAM50 classification up to 120 months post-surgery. The log-rank test was used to evaluate statistical differences

A total of 102 patients met all the selection criteria. Samples for which 95GC assessment results could not be obtained owing to problems with RNA quality were excluded, and ultimately 75 patients were selected for analysis. We followed the method described in the study by Parker et al. [14] to determine the breast cancer subtype (luminal A, luminal B, HER2-enriched, basal, or normal-like) using the PAM50 classification technique (Fig. 1).

The relationships between the distribution of each clinical factor and the 95GC results in the analyzed patients are shown in Table 1. Histologic grade, Ki-67, and PAM50 exhibited a significant relationship with the 95GC result as evaluated by Fisher’s exact test. Patients included in the 95GC-L group had mostly grade 2 luminal A breast cancer, and only 8.5% of the patients had Ki-67 staining of ≥20%. In contrast, patients in the 95GC-H group presented with grade 3, luminal B subtype, and 28.6% presented with Ki-67 staining of ≥20%. The three cases of pathological HER2-positive breast cancer were included in the five cases of PAM50 HER2-positive breast cancer but, because of this, the results of the pathological examination and classification by PAM50 may not completely match. This is because PAM50 is a classification based on gene expression levels using microarray data, and the method is different from that of pathological examination.

**Table 1:** Relationship between clinicopathological factors and 95GC results

Factors		95GC-L (*N* = 47) N (%)	95GC-H (*N* = 28) N (%)	Total (*N* = 75) N (%)	*p*-value
Age	≤50 years	14 (29.8)	11 (39.3)	25 (33.3)	*p* = 0.453
	> 50 years	33 (70.2)	17 (60.7)	50 (66.7)	
T stage	T1	30 (63.8)	16 (57.1)	46 (61.3)	*p* = 0.628
	T2	17 (36.2)	12 (42.9)	29 (38.7)	
Histology	Ductal	43 (91.5)	28 (100.0)	71 (94.7)	*p* = 0.384
	Lobular	1 (2.1)	0 (0.0)	1 (1.3)	
	Other	3 (6.4)	0 (0.0)	3 (4.0)	
Histologic grade	1	8 (17.0)	2 (7.1)	10 (13.3)	***p*** **< 0.001**
	2	33 (70.2)	10 (35.7)	43 (57.3)	
	3	6 (12.8)	15 (53.6)	21 (28.0)	
	NA	0 (0.0)	1 (3.6)	1 (1.3)	
PR	Negative	9 (19.1)	7 (25.0)	16 (21.3)	*p* = 0.572
	Positive	38 (80.9)	21 (75.0)	59 (78.7)	
HER2	Negative	46 (97.9)	26 (92.9)	72 (96.0)	*p* = 0.552
	Positive	1 (2.1)	2 (7.1)	3 (4.0)	
Ki-67	< 20%	37 (78.7)	16 (57.1)	53 (70.7)	***p*** **= 0.024**
	≥20%	4 (8.5)	8 (28.6)	12 (16.0)	
	NA	6 (12.8)	4 (14.3)	10 (13.3)	
PAM50	Luminal A	22 (46.8)	0 (0.0)	22 (29.3)	***p*** **< 0.001**
	Luminal B	7 (14.9)	16 (57.1)	23 (30.7)	
	HER2	1 (2.1)	4 (14.3)	5 (6.7)	
	Basal	2 (4.3)	8 (28.6)	10 (13.3)	
	Normal	15 (31.9)	0 (0.0)	15 (20.0)	

*NA* not available

### Prognostic performance of 95GC

**Fig. 2:**
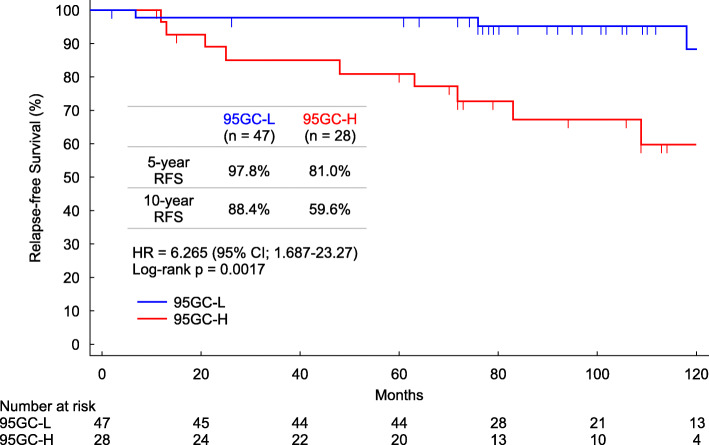
Evaluation of 95GC results and relapse outcome. Relapse-free survival was evaluated in patients included in the 95GC-L group (in blue) and 95GC-H group (in red) for up to 120 months post-surgery. The log-rank test was used to evaluate statistical differences

**Fig. 3:**
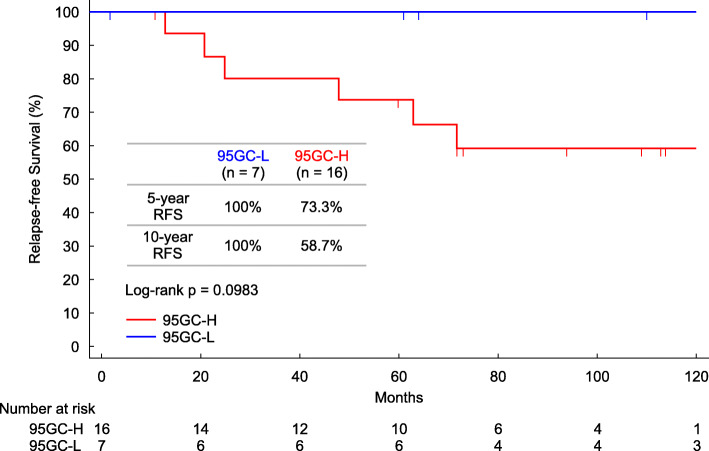
Relapse-free survival curves for PAM50 Luminal B for each result of 95GC. Relapse-free survival was evaluated in patients with luminal B tumors in the 95GC-L group (in blue) and 95GC-H group (in red) for up to 120 months post-surgery. The log-rank test was used to evaluate statistical differences

The relapse-free survival between the 95GC-L and 95GC-H groups showed a statistically significant difference (*p* = 0.0017). The 5-year relapse-free survival rates of the analyzed patients were 97.8% for the 95GC-L group and 81.0% for the 95GC-H group. The 10-year relapse-free survival rates were 88.4% for the 95GC-L group and 59.6% for the 95GC-H group (Fig. 2). Furthermore, luminal B tumors could be divided into 95GC-L and 95GC-H based on 95GC, with more favorable outcomes for 95GC-L than for 95GC-H (Fig. 3).

Univariate Cox regression analysis showed that Ki-67 and 95GC were the factors with significant hazard ratios for recurrence, whereas multivariate Cox regression analysis using these two factors showed that 95GC was a significant factor (Table 2). The three HER2-positive patients showed no recurrence; therefore, the hazard ratio was difficult to calculate and is not presented.

**Table 2:** Univariate and multivariate analyses of 95GC and other clinicopathological factors

Variable		Univariable analysis			Multivariable analysis		
		HR	95% CI	P	HR	95% CI	P
Age	≤50 vs > 50 years	0.60	0.19–1.90	0.3845			
T stage	T2 vs T1	1.44	0.45–4.55	0.5385			
Histologic grade	2 and 3 v 1	2.03	0.26–15.89	0.4986			
PR	Positive vs Negative	0.46	0.14–1.53	0.2055			
Ki-67	≥20% vs < 20%	3.69	1.16–11.67	0.0265	1.73	0.49–6.14	0.3982
PAM50	Others vs luminal A	5.74	0.74–44.56	0.0945			
95GC	95GC-H vs 95GC-L	6.26	1.69–23.27	0.0061	5.14	1.24–21.37	0.0242

### Association with histologic grade

**Fig. 4:**
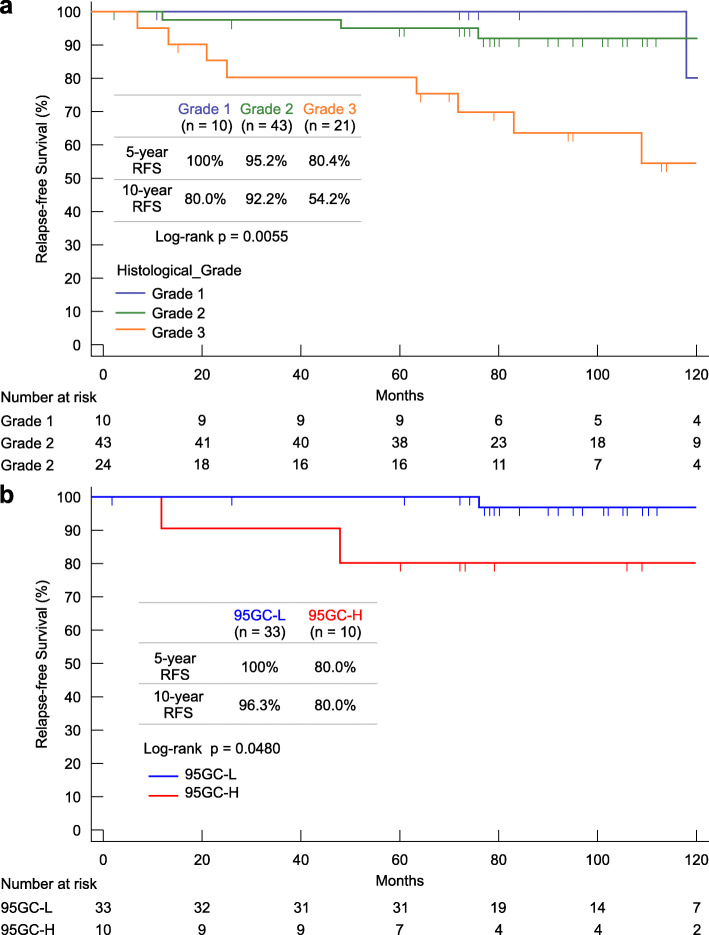
Relapse-free survival curves. **a** Relapse-free survival curves by histologic grade. **b** Relapse-free survival curves by 95GC result for histologic grade 2. Log-rank test was used to evaluate the statistical difference

Relapse-free survival rates correlated with the histologic grade (Fig. 4a). Furthermore, the relapse-free survival for the grade 2 subgroup was significantly different (*p* = 0.0480) between the 95GC-L and 95GC-H groups (Fig. 4b).

## Discussion

Although breast cancer outcomes have improved owing to recent advances in adjuvant chemotherapy, it is possible that patients who do not require chemotherapy also receive superfluous treatment. From this perspective, the possibility of separately evaluating the appropriateness of adjuvant chemotherapy for individual patients using multigene assays has garnered attention. The 95GC multigene assay is useful for the prognostic assessment of ER-positive, node-negative breast cancer [12, 13, 15]. However, previous studies regarding the usefulness of 95GC as a prognostic factor have been based only on patients from a single facility or public databases.

In the present investigation, we performed a retrospective study of the usefulness of 95GC in patients from five Japanese facilities with ER-positive, node-negative breast cancer who received endocrine therapy alone as adjuvant therapy. The 10-year relapse-free survival rate for patients diagnosed with 95GC-L was 88.4%, suggesting that favorable outcomes can be expected for these patients even if chemotherapy is omitted and only endocrine therapy is administered.

We examined the relationship between 95GC and intrinsic subtypes. The proportion of luminal A tumors was significantly higher among 95GC-L patients than among 95GC-H patients, whereas that of luminal B tumors was lower. Furthermore, luminal B tumors can be divided based on 95GC, with 95GC-L showing more favorable outcomes than 95GC-H. This finding demonstrates the possibility that patients with tumors classified by PAM50 as luminal B include a few for whom the addition of chemotherapy will have little beneficial effect on the outcome and that unnecessary chemotherapy for such patients may be reduced by combining PAM50 and 95GC results.

With regard to the association with histologic grade, when 95GC was used to divide the patients with grade 2 tumors into two groups (95GC-L and 95GC-H), the 10-year relapse-free survival rate for 95GC-L was 96.3%, which was significantly better than that for 95GC-H, and the relapse rate was extremely low. Therefore, chemotherapy can be considered omissible in patients with a histologic grade of 2 if they are 95GC-L.

By performing a multivariate analysis of 95GC and clinicopathological factors utilized in previous studies to assess breast cancer recurrence risk, such as age, tumor diameter, tumor grade, Ki-67, and PAM50, we were able to confirm that 95GC is a significant independent prognostic factor that influences the recurrence risk. In our cohort we did not include patients that have received neoadjuvant therapy. Because neoadjuvant chemotherapy is often added in tumors ≥3 cm, the T2 cohort included only cases with a size close to T1 stage, and as a result, there was no difference in prognosis between T1 and T2 stages.

The 95GC assay could provide extremely useful information for the selection of therapeutic strategies, such as adjuvant chemotherapy. The 95GC-L group had a better prognosis in response to adjuvant endocrine therapy than the 95GC-H group. Further prospective studies are needed to evaluate whether 95GC assays could be used to determine the necessity of chemotherapy in patients with ER-positive, node-negative breast cancer.

The Oncotype DX®, a breast cancer multigene assay, analyzes 21 genes using reverse transcription polymerase chain reaction, and classifies patients by recurrence score (RS) (0–100) into low-, mid-, and high-RS groups. It is a useful prognostic factor in hormone receptor-positive, HER2-negative, node-negative breast cancer and a factor for predicting the effectiveness of adjuvant chemotherapy in the high-RS group [5, 6]. Naoi et al. reported that in an indirect comparison with Oncotype DX® using Recurrence Online, 95GC could be used to classify 81 patients as having a mid-range risk (95GC-L group of 38 patients and 95GC-H group of 43 patients), with a significantly better recurrence-free survival rate in the 95GC-L group than in the 95GC-H group [13].

In everyday clinical practice, if a multigene assay is not available, Ki-67 is often used as a prognostic factor for ER-positive, HER2-negative breast cancer. In our study, Ki-67 measurement was performed through a central pathology review, but it was not a significant prognostic factor. It is difficult to establish a standardized method for evaluating Ki-67; therefore, it is difficult to accurately assess recurrence risk based on this factor. In the future, multigene assays for breast cancer should be incorporated more widely in everyday clinical practice.

With regard to the usefulness of 95GC as a predictive factor for the effectiveness of chemotherapy, Tsunashima et al. examined 72 patients from their facility and 287 public database patients who underwent neoadjuvant chemotherapy. They reported a significantly greater tumor-shrinking effect in the 95GC-H group than in the 95GC-L group [16]. Even though further prospective studies are needed, in the future, 95GC might be used not only as a prognostic factor but also as a predictive factor for the effectiveness of chemotherapy.

The main limitation of this study was its small sample size. Additionally, the retrospective nature of the study resulted in a selection bias. Moreover, because of the archived samples, the quality of which was not controlled owing to the surgical practices used in the earlier days, 26.5% (27/102) of the samples had poor RNA quality. However, in recent times, the strict rules enforced for the handling of post-extraction specimens have helped to preserve RNA quality and, thus, guarantee a high rate of sample recuperation in everyday clinical practice. Finally, all study participants were Japanese, and the performance when targeting other races in other countries is unknown. However, a recently published study evaluated the recurrence stratification of patients from both Japan and the US, with comparable results between populations [17].

Currently, 95GC has been available in Japan since 2013 for research purposes. Hence, further validation of the benefits of 95GC through a prospective study using a larger sample size is warranted to confirm the validity of incorporating 95GC in everyday clinical practice in Japan.

## Conclusions

This retrospective study suggests that 95GC is a long-term prognostic factor for ER-positive, node-negative breast cancer. To more optimally treat breast cancer patients, a system must be established to accurately assess the patient’s prognosis and determine whether the addition of chemotherapy will likely provide beneficial effects.

## Supplementary Information

**Additional file 1.** Strobe checklist.

## Data Availability

The datasets generated and/or analyzed during the current study are available from the corresponding author upon reasonable request.

## Abbreviations

ER: Estrogen receptor
95GC: Curebest™ 95GC Breast
RS: Recurrence score

## References

[1] Allemani C, Matsuda T, Di Carlo V, Harewood R, Matz M, Nikšić M (2018). Global surveillance of trends in cancer survival 2000-14 (CONCORD-3): analysis of individual records for 37 513 025 patients diagnosed with one of 18 cancers from 322 population-based registries in 71 countries. Lancet..

[2] Park S, Koo JS, Kim MS, Park HS, Lee JS, Lee JS, Kim SI, Park BW (2012). Characteristics and outcomes according to molecular subtypes of breast cancer as classified by a panel of four biomarkers using immunohistochemistry. Breast..

[3] Holleczek B, Stegmaier C, Radosa JC, Solomayer EF, Brenner H (2019). Risk of loco-regional recurrence and distant metastases of patients with invasive breast cancer up to ten years after diagnosis-results from a registry-based study from Germany. BMC Cancer.

[4] Wapnir IL, Price KN, Anderson SJ, Robidoux A, Martín M, Nortier JWR, Paterson AHG, Rimawi MF, Láng I, Baena-Cañada JM, Thürlimann B, Mamounas EP, Geyer CE, Gelber S, Coates AS, Gelber RD, Rastogi P, Regan MM, Wolmark N, Aebi S, on behalf of the International Breast Cancer Study Group, NRG Oncology, GEICAM Spanish Breast Cancer Group, BOOG Dutch Breast Cancer Trialists' Group, Breast International Group (2018). Efficacy of chemotherapy for ER-negative and ER-positive isolated locoregional recurrence of breast cancer: final analysis of the CALOR trial. J Clin Onc.

[5] Paik S, Shak S, Tang G, Kim C, Baker J, Cronin M, Baehner FL, Walker MG, Watson D, Park T, Hiller W, Fisher ER, Wickerham DL, Bryant J, Wolmark N (2004). A multigene assay to predict recurrence of tamoxifen-treated, node-negative breast cancer. N Engl J Med.

[6] Paik S, Tang G, Shak S, Kim C, Baker J, Kim W, Cronin M, Baehner FL, Watson D, Bryant J, Costantino JP, Geyer CE, Wickerham DL, Wolmark N (2006). Gene expression and benefit of chemotherapy in women with node-negative, estrogen receptor-positive breast cancer. J Clin Oncol.

[7] Sparano JA, Gray RJ, Makower DF, Pritchard KI, Albain KS, Hayes DF, Geyer CE Jr, Dees EC, Goetz MP, Olson JA Jr, Lively T, Badve SS, Saphner TJ, Wagner LI, Whelan TJ, Ellis MJ, Paik S, Wood WC, Ravdin PM, Keane MM, Gomez Moreno HL, Reddy PS, Goggins TF, Mayer IA, Brufsky AM, Toppmeyer DL, Kaklamani VG, Berenberg JL, Abrams J, Sledge GW Jr (2018). Adjuvant chemotherapy guided by a 21-gene expression assay in breast cancer. N Engl J Med.

[8] Carlson JJ, Roth JA (2013). The impact of the Oncotype dx breast cancer assay in clinical practice: a systematic review and meta-analysis. Breast Cancer Res Treat.

[9] Nielsen TO, Parker JS, Leung S, Voduc D, Ebbert M, Vickery T (2010). A comparison of PAM50 intrinsic subtyping with immunohistochemistry and clinical prognostic factors in tamoxifen-treated estrogen receptor positive breast cancer. Clin Cancer Res.

[10] Gnant M, Filipits M, Greil R, Stoeger H, Rudas M, Bago-Horvath Z, Mlineritsch B, Kwasny W, Knauer M, Singer C, Jakesz R, Dubsky P, Fitzal F, Bartsch R, Steger G, Balic M, Ressler S, Cowens JW, Storhoff J, Ferree S, Schaper C, Liu S, Fesl C, Nielsen TO, Austrian Breast and Colorectal Cancer Study Group (2014). Predicting distant recurrence in receptor-positive breast cancer patients with limited clinicopathological risk: using the PAM50 risk of recurrence score in 1478 postmenopausal patients of the ABCSG-8 trial treated with adjuvant endocrine therapy alone. Ann Oncol.

[11] Bartlett JMS, Bayani J, Marshall A, Dunn JA, Campbell A, Cunningham C (2016). Comparing breast cancer multiparameter tests in the OPTIMA Prelim Trial: no test is more equal than the others. J Natl Cancer Inst.

[12] Naoi Y, Kishi K, Tanei T, Tsunashima R, Tominaga N, Baba Y, Kim SJ, Taguchi T, Tamaki Y, Noguchi S (2011). Development of 95-gene classifier as a powerful predictor of recurrences in node-negative and ER-positive breast cancer patients. Breast Cancer Res Treat.

[13] Naoi Y, Kishi K, Tsunashima R, Shimazu K, Shimomura A, Maruyama N, Shimoda M, Kagara N, Baba Y, Kim SJ, Noguchi S (2013). Comparison of efficacy of 95-gene and 21-gene classifier (Oncotype DX) for prediction of recurrence in ER-positive and node-negative breast cancer patients. Breast Cancer Res Treat.

[14] Parker JS, Mullins M, Cheang MCU, Leung S, Voduc D, Vickery T, Davies S, Fauron C, He X, Hu Z, Quackenbush JF, Stijleman IJ, Palazzo J, Marron JS, Nobel AB, Mardis E, Nielsen TO, Ellis MJ, Perou CM, Bernard PS (2009). Supervised risk predictor of breast cancer based on intrinsic subtypes. J Clin Oncol.

[15] Naoi Y, Saito Y, Kishi K, Shimoda M, Kagara N, Miyake T, Tanei T, Shimazu K, Kim SJ, Noguchi S (2019). Development of recurrence risk score using 95-gene classifier and its application to formalin-fixed paraffin-embedded tissues in ER-positive, HER2-negative and node-negative breast cancer. Oncol Rep.

[16] Tsunashima R, Naoi Y, Kishi K, Baba Y, Shimomura A, Maruyama N, Nakayama T, Shimazu K, Kim SJ, Tamaki Y, Noguchi S (2012). Estrogen receptor positive breast cancer identified by 95-gene classifier as at high risk for relapse shows better response to neoadjuvant chemotherapy. Cancer Lett.

[17] Fujii T, Masuda H, Cheng YC, Yang F, Sahin AA, Naoi Y, Matsunaga Y, Raghavendra A, Sinha AK, Fernandez JRE, James A, Yamagishi K, Matsushima T, Schuetz R, Tripathy D, Tada S, Jackson RS, Noguchi S, Nakamura S, Acoba JD, Ueno NT (2021). A 95-gene signature stratifies recurrence risk of invasive disease in ER-positive, HER2-negative, node-negative breast cancer with intermediate 21-gene signature recurrence scores. Breast Cancer Res Treat.

